# *GLUT4* gene rs5418 polymorphism is associated with increased coronary heart disease risk in a Uygur Chinese population

**DOI:** 10.1186/s12872-022-02630-9

**Published:** 2022-04-25

**Authors:** Fei Yu, Fen Liu, Xiao-Mei Li, Qian Zhao, Jun-Yi Luo, Jin-Yu Zhang, Yi-Ning Yang

**Affiliations:** 1 Xinjiang Key Laboratory of Cardiovascular Disease, Clinical Medical Research Institute, The First Affiliated Hospital of Xinjiang Medical University, Urumqi, 830054 People’s Republic of China; 2 Department of Cardiology, First Affiliated Hospital of Xinjiang Medical University, Urumqi, 830054 People’s Republic of China; 3 Rehabilitation Medicine Department, First Affiliated Hospital of Xinjiang Medical University, Urumqi, 830054 People’s Republic of China

**Keywords:** Coronary heart disease, Glucose transporter 4 gene, Single nucleotide polymorphism, Susceptibility gene

## Abstract

**Background:**

To explore possible associations between glucose transporter 4 (*GLUT4*) genetic polymorphisms in the patients with coronary heart disease (CHD) in Han and Uygur Chinese populations in Xinjiang, China.

**Methods:**

Two *GLUT4* polymorphisms (rs5418 and rs5435) were genotyped in 1262 Han (628 CHD patients and 634 healthy controls) and 896 Uyghur (397 CHD patients and 499 healthy controls) Chinese populations.

**Results:**

In the Han Chinese population, there were no significant differences in allelic or genotypic distribution of rs5418 and rs5435 between the CHD and control groups (all *P* > 0.05). However, in the Uygur population, there were significant differences in genotype and allele distributions for rs5418 between CHD and the control group (all *P* < 0.05). Binary Logistic regression analysis showed that carriers with the rs5418 A allele had a higher risk of CHD compared to carriers of the rs5418 G allele (OR = 1.33, 95% CI: 1.069–1.649, *P* = 0.01), after adjustment for gender, age, drinking and smoking behavior, hypertension and diabetes. Furthermore, haploid association analysis of the two SNP loci of the *GLUT4* gene showed that the AC haplotype was associated with CHD in the Uygur population (*P* = 0.001598; OR = 1.36, 95% CI = 1.1228–1.6406).

**Conclusions:**

rs5418 *GLUT4* gene variants are associated with CHD in the Uygur Chinese population.

**Supplementary Information:**

The online version contains supplementary material available at 10.1186/s12872-022-02630-9.

## Background

Coronary heart disease (CHD), also known as ischemic heart disease, is caused by myocardial ischemia and hypoxia due to changes in coronary circulation and represents the greatest mortality risk globally of all diseases. Prior reports suggest that ischemic heart disease causes 12.9 million deaths, accounting for one quarter of all global deaths annually, and is the main cause of death [1]. Many factors have been implicated in the development of coronary heart disease including smoking [2], obesity [3], sex [4], metabolic syndrome severity [5], glycerophosphocholine [6], dysglycemia and diabetes [7, 8], and sleep apnea syndrome [9, 10]. In addition to these environmental factors, several gene variants have been linked to the development and progression of CHD [11–13].

Glucose transporters (*GLUT*) are necessary for glucose uptake in cells of nearly all species [14]. Of the 14 known human *GLUT* genes, *GLUT 1, 2, 3*, and *4* have been implicated in human disease. In particular *GLUT4* has been the subject of significant study as it is the main glucose transporter found in muscles and adipocytes [14]. This 12-transmembrane, 509-amino acid protein contains a large cytoplasmic loop between transmembrane helices 6 and 7 that is normally sequestered intracellularly in the basal state [15]. The cellular localization of *GLUT4* is regulated by insulin through a complex mechanism, the aberration of which contributes to obesity [14]. The *GLUT4* gene (also known as the solute carrier family 2, member 4 (17p13.2; OMIM 138190) encodes the *GLUT4* solute carrier [16]. Many studies across multiple ethnicities have suggested a relationship between *GLUT4* polymorphisms and human disease including insulin resistance [17–19], type 2 diabetes, [20] and obstructive sleep apnea syndrome [21]. However, few studies have investigated the association between the *GLUT4* gene and CHD [22]. Thus, we aimed to evaluate a possible relationship between *GLUT4* gene polymorphisms and CHD risk in Han and Uygur populations in Xinjiang, China.

## Methods

### Subjects

In this case–control study, patients with coronary angiography were recruited from the cardiovascular department of the First Affiliated Hospital of Xinjiang Medical University. The inclusion criteria for CHD are as follows: (1) older than 18 years of age, (2) a diagnosis of CHD according to the World Health Organization (WHO) CHD diagnostic criteria set in 1979, and (3) single vessel stenosis of > 70%, or multiple vessel stenosis of > 50%. The controls consisted of a random sample of adult’s individuals above 18 years of age who were visiting the hospital for a routine checkup and had medical records. The inclusion criteria were being devoid from any type of cardiovascular diseases and having a normal coronary artery with no evidence of plaque or peripheral vascular disease as confirmed by angiography, medical profile, or previous medical history as documented in their medical records. Individuals were excluded if they had the following conditions: cancer, acute or chronic inflammatory disease, cardiomyopathy or heart valve disease, variant angina without fixed coronary stenosis, coronary artery dilatation, X syndrome, serious liver disease (ALT > 2X normal), serious kidney disease (blood CR > 2.5 mg/dl), blood disease and pregnancy.

### Clinical data collection

Baseline characteristics of all patients, such as sex, age, and smoking status were collected and are summarized in Table 1. Height and body mass were measured by a unified standard method, and patients' previous medical history such as hypertension, diabetes, abnormal blood lipid metabolism, and medications were recorded and used to diagnose related diseases in accordance with relevant Chinese or international standards [23, 24]. Habitual activities such as smoking and alcohol consumption behavior were collected from questionnaire.

**Table 1:** Baseline characteristic of the Han population

Characteristic	CHD (n = 628)	Controls (n = 634)	*P*
Sex (M/F)	448/180	290/344	< 0.001
Age, mean ± SD	58.91 ± 12.41	55.877 ± 10.19	< 0.001
BMI, mean ± SD	25.29 ± 3.25	24.98 ± 3.25	0.249
*Drinking*			
0	408	473	< 0.001
1	97	161	
2	123	0	
*Smoking*			
0	101	455	< 0.001
1	245	178	
2	6	0	
3	276	1	
*Hypertension*			
No	320	364	0.024
Yes	308	270	
*DM*			
No	463	572	< 0.001
Yes	165	62	

*SD* standard deviation, *BMI* body mass index; Among the smokers, 0 means never smoking, 1 means occasional smoking, smoking cigarettes more than 4 times a week, but less than 1 cigarette per day on average, 2 means regular smoking, smoking cigarettes more than 1 cigarette per day, continuous or accumulative for 6 months. 3 represents heavy smoking, smoking more than 2 cigarettes a day for more than 6 months. Zero for non-drinking, 1 for occasional drinking, consuming about 10 g of alcohol per serving, and 2 for regular drinking, exceeding about 10 g of alcohol

### Blood biochemical indexes and DNA extraction

Cubital venous blood was collected from subjects after fasting for > 12 h. Blood biochemical indexes were tested by the Medical Laboratory Center of the First Affiliated Hospital of Xinjiang Medical University using the Abbott C16000 biochemical immunity instrument (United States). The Whole Blood Genome Extraction Kit (Tiangen Biochemical Co., Ltd.) was used to extract DNA from whole blood samples. A Nanodrop ND-2000 Ultramicro Nucleic Acid Analyzer was used to detect the concentration and purity of DNA, which was then diluted to 20 ng/ul.

### Genotyping

In this study, single nucleotide polymorphisms (SNPs) were typed using the TM Multiple SNP Typing Kit (Shanghai Genesky Biotechnology). The SNP allelic site was identified using a high specificity ligase reaction. Then, the ligated products of different lengths were obtained by introducing varied length non-specific sequences at the end of the ligase probe and using a ligase addition reaction. The ligated products were PCR amplified using universal primers labeled with fluorescence, as shown in Supplementary material, and the amplified products were separated by fluorescence capillary electrophoresis. Finally, the genotypes of each SNP locus were obtained by analyzing the electrophoretic patterns.

### Data analyses

SPSS 20.0 software was used for statistical analysis. Student t-test is applied for the continuous variables, while Pearson chi-square test is for categorical variables. Wilcoxon Rank-Sum test was applied for body mass index (BMI). The software program SHEsis or chi-square test (http://analysis.bio-x.cn) was used to assess Hardy- Weinberg equilibrium (HWE). PLINK (http://pngu.mgh.harvard.edu/purcell/plink/) software was used to analyze the allelic and genotypic association with disease [25]. Linkage disequilibrium (LD) plot was generated from Haploview version 4.2 (Broad Institute, Cambridge, MA, United States) [26]. Haplotype association analyses were performed with PLINK. Binary logistic regression, with the adjustment of covariates was applied to calculate the odds ratios (ORs) and 95% confidence intervals (CIs). Three logistic regression models (additive, dominant, and recessive) were also used to analyze the SNPs. All tests were two-tailed, and the results were considered significant when *P* ≤ 0.05.

## Results

### Baseline characteristics

The clinical characteristics of study subjects in the Han and Uygur populations are shown in Tables 1 and 2, respectively. Significant differences between both populations were noted in regard to gender (*P* < 0.001), age (*P* < 0.001), drinking and smoking behavior (*P* < 0.001), hypertension (*P* < 0.001) and diabetes (*P* < 0.001). Accordingly, these variants were used as the covariates in the following binary logistic regression analysis. No statically significant difference in the BMI was found between CHD patients and controls.

**Table 2:** Baseline characteristic of the Uygur population

Characteristic	CHD (n = 397)	Controls (n = 499)	*P*
Sex (M/F)	280/117	244/255	< 0.001
Age, mean ± SD	55.864 ± 9.00	52.374 ± 9.29	< 0.001
BMI, mean ± SD	27.39 ± 3.65	27.26 ± 4.31	0.787
*Drinking*			
0	291	409	< 0.001
1	95	90	
2	11	0	
*Smoking*			
0	190	376	< 0.001
1	178	123	
2	1	0	
3	28	0	
*Hypertension*			
No	194	282	0.026
Yes	203	217	
*DM*			
No	291	432	< 0.001
Yes	106	67	

*SD* standard deviation, *BMI* body mass index; Among the smokers, 0 means never smoking, 1 means occasional smoking, smoking cigarettes more than 4 times a week, but less than 1 cigarette per day on average, 2 means regular smoking, smoking cigarettes more than 1 cigarette per day, continuous or accumulative for 6 months. 3 represents heavy smoking, smoking more than 2 cigarettes a day for more than 6 months. Zero for non-drinking, 1 for occasional drinking, consuming about 10 g of alcohol per serving, and 2 for regular drinking, exceeding about 10 g of alcohol

### Hardy–Weinberg analysis of the investigated SNPs

The Hardy–Weinberg analysis showed no deviations from Hardy–Weinberg Equilibrium for rs5418 and rs5435 in either the Han or Uygur populations.

### Allelic and genotypic association with CHD

In the Han population, neither rs5418 nor rs5435 variants showed any significant differences in allelic frequencies and genotypic distribution between the CHD and control groups (all *P* > 0.05). However, in the Uygur population, the allelic frequencies of A and G at the rs5418 locus were 46.22 and 53.78% in the CHD group, and 39.28 and 60.72% in the control group, respectively, representing a significant difference in allelic distribution (*P* = 0.003, 95% CI = 1.10–1.61). The risk of having CHD in patients with the A allele was 1.33 times greater than that of the controls. The frequencies of the T and C alleles at the rs5435 locus in the Uygur CHD group were 33.12 and 66.88%, respectively, compared to 37.37 and 62.63% in the control group, respectively, representing no significant difference in the distribution of the alleles and genotypes between the two groups at the rs5435 locus (*P* = 0.06, OR = 0.83, 95% CI = 0.68–1.01). We conducted further binary logistic regressions based on the additive, dominant and recessive model after controlling for the influence of gender, age, drinking and smoking behavior, hypertension and diabetes, which revealed a significant association between the rs5418 locus and the risk of CHD under all the genetic models. Compared with the G allele, the A allele increased the risk of coronary heart disease 1.33×. These data are summaries in Tables 3, 4 and 5.

**Table 3:** Allelic association analysis between two SNPs and CHD

Population	SNP	Allele	Group		*χ*^2^	*P*	S.E	OR (95%CI)
			CHD group (%)	Control group (%)				
Han	rs5418	A	488 (38.85)	495 (39.04)	0.01	0.92	0.08	0.99 (0.85–1.16)
		G	768 (61.15)	773 (60.96)				
	rs5435	T	397 (31.61)	406 (32.02)	0.05	0.82	0.09	0.98 (0.83–1.16)
		C	859 (68.39)	862 (67.98)				
Uygur	rs5418	A	367 (46.22)	392 (39.28)	8.73	0.003	0.10	1.33 (1.10–1.61)
		G	427 (53.78)	606 (60.72)				
	rs5435	T	263 (33.12)	373 (37.37)	3.49	0.06	0.10	0.83 (0.68–1.01)
		C	531 (66.88)	625 (62.63)

**Table 4:** Genotypic association analysis between two SNPs and CHD

Population	SNP	Genotype	CHD group (%)	Control group (%)	OR(95%CI)(*P*)		
					Dominant model	Additive model	Recessive model
Han	rs5418	AA	99 (15.76)	97 (15.30)	0.97 (0.77–1.21) (0.76)	Ref	1.04 (0.76–1.41) (0.82)
		AG	290 (46.18)	301 (47.48)		1.06 (0.77–1.46) (0.73)	
		GG	239 (38.06)	236 (37.22)		1.01 (0.72–1.41) (0.96)	
	rs5435	TT	59 (9.39)	63 (9.94)	0.99 (0.79–1.23) (0.92)	Ref	0.94 (0.65–1.37) (0.75)
		TC	279 (44.43)	280 (44.16)		0.94 (0.64–1.39) (0.76)	
		CC	290 (46.18)	291 (45.90)		0.94 (0.64–1.39) (0.76)	
Uygur	rs5418	AA	82 (20.66)	67 (13.43)	1.36 (1.02–1.81) (0.03)	Ref	1.68 (1.18–2.39) (0.004)
		AG	203 (51.13)	258 (51.70)		1.56 (1.07–2.26) (0.020)	
		GG	112 (28.21)	174 (34.87)		1.90 (1.27–2.84) (0.0017)	
	rs5435	TT	43 (10.84)	66 (13.23)	0.78 (0.59–1.02) (0.06)	Ref	0.80 (0.53–1.20) (0.28)
		TC	177 (44.58)	241 (48.30)		0.89 (0.58–1.36) (0.59)	
		CC	177 (44.58)	192 (38.47)		0.71 (0.46–1.09) (0.12)

**Table 5:** Logistic regression analysis of GLUT4 polymorphisms and risk of coronary heart disease in the Uygur population

SNP	Allele	Test	OR (95% CI)	*P*
rs5418	A–G	Additive model	1.33 (1.07–1.65)	0.010
		Dominant model	1.36 (0.99–1.87)	0.053
		Recessive model	1.59 (1.07–2.36)	0.022
rs5435	T–C	Additive model	0.83 (0.67–1.03)	0.089
		Dominant model	0.74 (0.55–0.99)	0.048
		Recessive model	0.89 (0.57–1.28)	0.590

For rs5418, genotype AA, AG and GG is coded as 0, 1 and 2 in additive model; AA, AG is coded as 0 and GG is coded as 1 in dominant model; AA is coded as 0 and AG, GG is coded as 1 in recessive model. For rs5435, genotype TT, TC and CC is coded as 0, 1 and 2 in additive model; TT, TC is coded as 0 and CC is coded as 1 in dominant model; TT is coded as 0 and TC, CC is coded as 1 in recessive model

We explored the correlations between rs5418 and other risk factors. There are no association identified between rs5418 and most of the risk factors. In Uygur population, genotype of rs5418 was significantly correlated with smoking behavior (Additional file 1: Tables S1, S2).

### Haplotypes associated with CHD

Additional file 2: Figure S1 was included to show the linkage disequilibrium for the two investigated SNPs. In the Han population, haploid frequency analysis of two *GLUT4* SNP loci identified three haplotypes at the two loci, yet there was no significant difference in the distribution of these haplotypes in the Han population (*P* > 0.05). In the Uygur population, haploid frequency analysis of two *GLUT4* SNP loci identified four haplotypes. In particular, AC haplotype distribution was significantly different between the CHD and control groups in the Uygur population (*P* < 0.05) and the risk of developing CHD increased 1.36 for the AC haplotype carriers (Table 6).

**Table 6:** Association of haplotypes with CHD

Haplotype (Han)	CHD group n = 628	Control group n = 634	*χ*^*2*^	*P*	OR (95% CI)	SNPs
GT	390	400	0.097	0.76	0.98 (0.83–1.16)	rs5418|rs5435
AC	481	490	0.03	0.86	0.99 (0.84–1.16)	rs5418|rs5435
GC	385	377	0.25	0.62	1.04 (0.88–1.24)	rs5418|rs5435

Haplotype (Uygur)	CHD group n = 397	Control group n = 499	*χ*^*2*^	*P*	OR (95% CI)	SNPs
AT	10	17	0.57	0.45	0.74(0.34–1.62)	rs5418|rs5435
GT	253	356	2.87	0.090	0.84 (0.69–1.03)	rs5418|rs5435
AC	357	375	9.96	0.0016	1.36 (1.12–1.64)	rs5418|rs5435
GC	174	250	2.40	0.12	0.84 (0.67–1.05)	rs5418|rs5435

## Discussion

Previous studies have shown close relationships between the incidence of CHD and *LAP* (liver-enriched transcriptional activator protein), *(IL)-1* (interleukin 1), *IL-18* (interleukin 18), *ANGPTL4* (Angiopoietin-like 4), and *PIN1* (Peptidylprolyl Cis/Trans Isomerase, NIMA-Interacting 1) [27–31] but few studies have investigated the association between *GLUT4* polymorphisms and CHD. We studied *GLUT4* polymorphisms and potential associations with CHD in multiple ethnic groups within a Chines population and identified a specific *GLUT4* gene polymorphism that is significantly associated with the risk of developing CHD in the Uygur Chinese population.

*GLUT4* is the primary glucose transporter in the human heart, which accounts for approximately 70% of all glucose transport [32]. In heart diseases like cardiac hypertrophy, heart failure, and myocardial ischemia different perturbations in expression of glucose transporters are observed, especially in *GLUT1* and *GLUT4*, due to changes in heart glucose metabolism. *GLUT4* is translocated to the cell surface in response to ischemia, insulin, and catecholamine*s*, allowing it to increase glucose transport into cardiomyocytes. However, in the absence of stimulation, *GLUT4* is trapped intracellularly [33]. Ischemia, as seen in CHD, results in increased myocardial glucose utilization derived from glycogen breakdown [34] and also leads to increased glucose transport activity [35]. The increase in glucose uptake is due to the transport of *GLUT1* and *GLUT4* from the intracellular ventricle to the sarcolemma. Many studies have reported *GLUT4* gene polymorphisms in association with type 2 diabetes [20, 36–38], but there are few investigations of possible *GLUT4* gene polymorphisms in CHD patients.

Of the known *GLUT4* polymorphisms, rs5418 is located in the gene promoter region [39]. One prior study in a south India population found that the *GLUT4* gene was detected in normal glucose tolerance and type 2 diabetes, and found differences in the ACGT haplotype of rs5418 [20]. Another study found that the rs5418 locus is associated with HBA1c levels in Japanese males [40]. The rs5418 locus has also been linked with obesity despite a similar allelic and genotypic distribution in extremely obese children and adolescents compared to normal and underweight patients, suggesting that these alleles are not involved in weight regulation [41]. The rs5435 locus was reported associated with risk of CHD in Han population in Guangdong province [22]. However, no significant association with CHD in Han and Uygur population in our study in allice and genotype distribution. The reason might be low sample size decreased the statistical power, since a significant trend was identified in logistic model under dominant model.

In this study, we found that the genotypic and allelic frequencies of rs5418 locus were not significantly different in either the CHD or the control group of a Han Chinese population. However, in the Uygur population, we found that the distribution frequencies of the three genotypes and alleles in the two groups were significantly different. Differences in findings between these populations are likely related to the races studied. As a multi-ethnic region, Xinjiang has 45 ethnic groups, in addition to the Han and Uygur populations. In addition to the different genetic backgrounds of these groups, there are a variety of cultural and lifestyle differences that may also play a contributing role. Our findings that the AA genotype and the A allele at the rs5418 site *GLUT4* gene are associated with susceptibility to CHD in the Uygur Chinese population, it may be beneficial provide for future clinical diagnosis and treatment. This study was limited to only patients from two ethnic groups in Xinjiang, China. Future studies should include more ethnic groups from this area or study populations in other regions of China or worldwide. Further functional validation of increased susceptibility to CHD development is also needed to confirm a definitive role of rs5418 site mutations in the *GLUT4* gene and elucidate the effects of these mutations on CHD pathogenesis or etiology.

## Conclusions

*GLUT4* gene polymorphisms at the rs5418 site were associated with CHD susceptibility in the Uygur, but not Han, Chinese population of Xinjiang, China.

## Supplementary Information

**Additional file 1: Table S1.** Baseline characteristic of the Han population according to the genotype of rs5418. **Table S2.** Baseline characteristic of the Uygur population according to the genotype of rs5418.

**Additional file 2: Figure S1.** Haploview analysis for D' and r2 pairwise measures of LD between rs5418 and rs5435. D' values and confidence levels (LOD) are represented as black for D' = 1, LOD > 2; shades of pink for high D', LOD < 2; white for D' < 1, LOD < 2. r2 values are represented as black for r2 = 1, white for r2 = 0, with intermediate values for 0 < r2 < 1 indicated by shades of grey. The numbers within the squares represent the D' or r2 scores for pairwise LD.

## Data Availability

The datasets of genotyping data in the current study are available as the PLINK binary format in the Github repository [https://github.com/2022yufei/genotyping].

## Abbreviations

CHD: Coronary heart disease
*GLUT4*: Glucose transporter 4 gene
SNPs: Single nucleotide polymorphisms
PCR: Polymerase chain reaction
HWE: Hardy–Weinberg equilibrium
OR: Odds ratio

## References

[1] Lozano R, Naghavi M, Foreman K, Lim S, Shibuya K, Aboyans V (2012). Global and regional mortality from 235 causes of death for 20 age groups in 1990 and 2010: a systematic analysis for the Global Burden of Disease Study 2010. Lancet.

[2] Khoramdad M, Vahedian-Azimi A, Karimi L, Rahimi-Bashar F, Amini H, Sahebkar AJ (2020). Association between passive smoking and cardiovascular disease: a systematic review and meta-analysis. IUBMB Life.

[3] Chirinos D, Llabre M, Goldberg R, Gellman M, Mendez A, Cai J (2020). Defining abdominal obesity as a risk factor for coronary heart disease in the U.S.: results from the Hispanic Community Health Study/Study of Latinos (HCHS/SOL). Diabetes Care.

[4] Bancks M, Akhabue E, Rana J, Reis JP, Schreiner PJ, Yano Y (2020). Sex differences in cardiovascular risk factors before and after the development of type 2 diabetes and risk for incident cardiovascular disease. Diabetes Res Clin Pract.

[5] DeBoer M, Gurka M, Golden S, Musani SK, Sims M, Vishnu A (2017). Independent associations between metabolic syndrome severity and future coronary heart disease by sex and race. J Am Coll Cardiol.

[6] Syme C, Czajkowski S, Shin J, Abrahamowicz M, Leonard G, Perron M (2016). Glycerophosphocholine metabolites and cardiovascular disease risk factors in adolescents: a cohort study. Circulation.

[7] Ross S, Gerstein H, Eikelboom J, Anand S, Yusuf S, Paré G (2015). Mendelian randomization analysis supports the causal role of dysglycaemia and diabetes in the risk of coronary artery disease. Eur Heart J.

[8] Laakso M, Kuusisto J (2014). Insulin resistance and hyperglycaemia in cardiovascular disease development. Nat Rev Endocrinol.

[9] Lee C, Sethi R, Li R, Ho HH, Hein T, Jim MH (2016). Obstructive sleep apnea and cardiovascular events after percutaneous coronary intervention. Circulation.

[10] Gami A, Olson E, Shen W, Wright RS, Ballman KV, Hodge DO (2013). Obstructive sleep apnea and the risk of sudden cardiac death: a longitudinal study of 10,701 adults. J Am Coll Cardiol.

[11] Kessler T, Schunkert H (2021). coronary artery disease genetics enlightened by genome-wide association studies. JACC Basic Transl Sci.

[12] Nikpay M, Goel A, Won HH, Hall LM, Willenborg C, Kanoni S (2015). A comprehensive 1000 Genomes–based genome-wide association meta-analysis of coronary artery disease. Nat Genet.

[13] Matsunaga H, Ito K, Akiyama M, Takahashi A, Koyama S, Nomura S (2020). Transethnic meta-analysis of genome-wide association studies identifies three new loci and characterizes population-specific differences for coronary artery disease. Circul Genomic Precis Med.

[14] Sun L, Zeng X, Yan C, Sun X, Gong X, Rao Y (2012). Crystal structure of a bacterial homologue of glucose transporters GLUT1-4. Nature.

[15] Mishra R, Wei C, Hresko R, Bajpai R, Heitmeier M, Matulis SM (2015). In silico modeling-based identification of glucose transporter 4 (GLUT4)-selective inhibitors for cancer therapy. J Biol Chem.

[16] Al-Lahham Y, Mendes A, Souza E, Alberton D, Rego F, Valdameri G, et al. Interleukin-18 (rs187238) and glucose transporter 4 (rs5435) polymorphisms in Euro-Brazilians with type 1 diabetes. Genet Mol Res. 2017;16(3). 10.4238/gmr16039755 28973736

[17] Zisman A, Peroni O, Abel E, Michael MD, Mauvais-Jarvis F, Lowell BB (2000). Targeted disruption of the glucose transporter 4 selectively in muscle causes insulin resistance and glucose intolerance. Nat Med.

[18] Abel E, Peroni O, Kim J, Kim YB, Boss O, Hadro E (2001). Adipose-selective targeting of the GLUT4 gene impairs insulin action in muscle and liver. Nature.

[19] Jiang L, Yao L, Yang Y, Ke D, Batey R, Wang J (2016). Jiangzhi Capsule improves fructose-induced insulin resistance in rats: Association with repair of the impaired sarcolemmal glucose transporter-4 recycling. J Ethnopharmacol.

[20] Bodhini D, Radha V, Ghosh S, Majumder PP, Rao MR, Mohan V (2011). GLUT4 gene polymorphisms and their association with type 2 diabetes in south Indians. Diabetes Technol Ther.

[21] Yin T, Li N, Ai L, Yao X, Hong J, Zhou L (2014). Association of glucose transporter 4 gene polymorphism with hypoxia caused by obstructive sleep apnea syndrome and with related inflammatory factors. Zhong guo Yi Xue Ke Xue Yuan Xue Bao.

[22] Xiang Z, Lu J, Yeda C, Meihua L, Jiheng Q (2016). Association of GLUT4 gene polymorphisms with risk of coronary heart disease in Han population in Guangdong province. Chin J Public Health.

[23] Zhong M, Tan H, Gong H, Wang S, Zhang Y, Zhang W (2008). Increased serum visfatin in patients with metabolic syndrome and carotid atherosclerosis. Clin Endocrinol (Oxf).

[24] Johansson L, Johansson L, Ridderstrale M (2008). The visfatin (PBEF1) G-948T gene polymorphism is associated with increased high-density lipoprotein cholesterol in obese subjects. Metabolism.

[25] Purcell S, Neale B, Todd-Brown K, Thomas L, Ferreira MA, Bender D (2007). PLINK: a tool set for whole-genome association and population-based linkage analyses. Am J Hum Genet.

[26] Belfer I, Buzas B, Evans C, Hipp H, Phillips G, Taubman J (2005). Haplotype structure of the beta adrenergic receptor genes in US Caucasians and African Americans. Eur J Hum Genet.

[27] Wei W, Li X, Feng Q, Kubo M, Kullo I, Peissig P (2018). LPA variants are associated with residual cardiovascular risk in patients receiving statins. Circulation.

[28] Tsimikas S, Duff G, Berger P, Rogus J, Huttner K, Clopton P (2014). Pro-inflammatory interleukin-1 genotypes potentiate the risk of coronary artery disease and cardiovascular events mediated by oxidized phospholipids and lipoprotein(a). J Am Coll Cardiol.

[29] Tiret L, Godefroy T, Lubos E, Nicaud V, Tregouet D, Barbaux S (2005). Genetic analysis of the interleukin-18 system highlights the role of the interleukin-18 gene in cardiovascular disease. Circulation.

[30] Stitziel N, Stirrups K, Masca N, Erdmann J, Ferrario PG, König IR (2016). Coding variation in ANGPTL4, LPL, and SVEP1 and the risk of coronary disease. N Engl J Med.

[31] Wang J, Du W, Bai J, Cheng S, Zhang YH (2020). Association cdtojoNS: The association of rs2233679 in the PIN1 gene promoter with the risk of Coronary Artery Disease in Chinese female individuals. J Stroke Cerebrovasc Dis.

[32] Mueckler M, Thorens B (2013). The SLC2 (GLUT) family of membrane transporters. Mol Aspects Med.

[33] Szablewski L (2017). Glucose transporters in healthy heart and in cardiac disease. Int J Cardiol.

[34] Depré C, Rider M, Hue L (1998). Mechanisms of control of heart glycolysis. Eur J Biochem.

[35] Young L, Coven D, Russell RR (2000). Cellular and molecular regulation of cardiac glucose transport. J Nucl Cardiol.

[36] Al-Lahham Y, Mendes A, Souza E, Alberton D, Rego F, Valdameri G,et al. Interleukin-18 (rs187238) and glucose transporter 4 (rs5435) polymorphisms in Euro-Brazilians with type 1 diabetes. Genet Mol Res. 2017;16(3). 10.4238/gmr16039755 28973736

[37] Pontiroli A, Capra F, Veglia F, Ferrari M, Xiang K, Bell G (1996). Genetic contribution of polymorphism of the GLUT1 and GLUT4 genes to the susceptibility to type 2 (non-insulin-dependent) diabetes mellitus in different populations. Acta Diabetol.

[38] Alcolado J, Baroni M (1992). Restriction fragment length polymorphisms at the GLUT4 and GLUT1 gene loci in type 2 diabetes. Diabet Med J Br Diabet Assoc.

[39] Bjørbaek C, Echwald S, Hubricht P, Vestergaard H, Hansen T, Zierath J (1994). Genetic variants in promoters and coding regions of the muscle glycogen synthase and the insulin-responsive GLUT4 genes in NIDDM. Diabetes.

[40] Xi C, Miyaki K, Ikeda S, Song Y, Sinbo T, Muramatsu M (2012). Association of GLUT4 gene variants with HbA1c level in Japanese men. Endocr J.

[41] Friedel S, Antwerpen B, Hoch A, Vogel C, Grassl W, Geller F (2002). Glucose transporter 4 gene: association studies pertaining to alleles of two polymorphisms in extremely obese children and adolescents and in normal and underweight controls. Ann N Y Acad Sci.

