# Supplementary material for: GLUT4 gene rs5418 polymorphism is associated with increased coronary heart disease risk in a Uygur Chinese population
Source: BMC Cardiovasc Disord. 2022 Apr 25;22:191. doi: 10.1186/s12872-022-02630-9 (PMC9036804; doi:10.1186/s12872-022-02630-9)
Supplement: Supplementary file 1 — Additional file 1: Table S1. Baseline characteristic of the Han population according to the genotype of rs5418. Table S2. Baseline characteristic of the Uygur population according to the genotype of rs5418. [file 12872_2022_2630_MOESM1_ESM.docx]

Table S1. Baseline characteristic of the Han population according to the genotype of rs5418

| Characteristics | A/A (N=196) | G/A (N=591) | G/G (N=475) | Total(N=1262) | pvalue |
| --- | --- | --- | --- | --- | --- |
| Sex |  |  |  |  | 0.48 |
| Male | 112(8.87%) | 338(26.78%) | 288(22.82%) | 738(58.48%) |  |
| Female | 84(6.66%) | 253(20.05%) | 187(14.82%) | 524(41.52%) |  |
| Age |  |  |  |  |  |
|  | 57.83±11.69 | 57.47±11.39 | 57.09±11.44 | 57.39±11.45 | 0.73 |
| Smoking |  |  |  |  | 0.54 |
| 0 | 87(6.89%) | 272(21.55%) | 197(15.61%) | 556(44.06%) |  |
| 1 | 67(5.31%) | 196(15.53%) | 160(12.68%) | 423(33.52%) |  |
| 2 | 1(0.08%) | 4(0.32%) | 1(0.08%) | 6(0.48%) |  |
| 3 | 41(3.25%) | 119(9.43%) | 117(9.27%) | 277(21.95%) |  |
| Drinking |  |  |  |  | 0.75 |
| 0 | 132(10.46%) | 423(33.52%) | 326(25.83%) | 881(69.81%) |  |
| 1 | 44(3.49%) | 112(8.87%) | 102(8.08%) | 258(20.44%) |  |
| 2 | 20(1.58%) | 56(4.44%) | 47(3.72%) | 123(9.75%) |  |
| Hypertension |  |  |  |  | 0.84 |
| No | 109(8.64%) | 322(25.52%) | 253(20.05%) | 684(54.20%) |  |
| Yes | 87(6.89%) | 269(21.32%) | 222(17.59%) | 578(45.80%) |  |
| DM |  |  |  |  | 0.24 |
| No | 161(12.76%) | 474(37.56%) | 400(31.70%) | 1035(82.01%) |  |
| Yes | 35(2.77%) | 117(9.27%) | 75(5.94%) | 227(17.99%) |  |

Table S2. Baseline characteristic of the Uygur population according to the genotype of rs5418

| Characteristics | A/A (N=149) | A/G (N=461) | G/G (N=286) | Total(N=896) | pvalue |
| --- | --- | --- | --- | --- | --- |
| Sex |  |  |  |  | 0.22 |
| Male | 96(10.71%) | 260(29.02%) | 168(18.75%) | 524(58.48%) |  |
| Female | 53(5.92%) | 201(22.43%) | 118(13.17%) | 372(41.52%) |  |
| Age |  |  |  |  |  |
|  | 54.46±9.01 | 53.63±9.40 | 54.11±9.36 | 53.92±9.32 | 0.59 |
| Smoking |  |  |  |  | 1.2e-3 |
| 0 | 93(10.38%) | 287(32.03%) | 186(20.76%) | 566(63.17%) |  |
| 1 | 44(4.91%) | 158(17.63%) | 99(11.05%) | 301(33.59%) |  |
| 2 | 1(0.11%) | 0(0.0e+0%) | 0(0.0e+0%) | 1(0.11%) |  |
| 3 | 11(1.23%) | 16(1.79%) | 1(0.11%) | 28(3.13%) |  |
| Drinking |  |  |  |  | 0.58 |
| 0 | 117(13.06%) | 359(40.07%) | 224(25.00%) | 700(78.13%) |  |
| 1 | 30(3.35%) | 94(10.49%) | 61(6.81%) | 185(20.65%) |  |
| 2 | 2(0.22%) | 8(0.89%) | 1(0.11%) | 11(1.23%) |  |
| Hypertension |  |  |  |  | 0.91 |
| No | 80(8.93%) | 247(27.57%) | 149(16.63%) | 476(53.13%) |  |
| Yes | 69(7.70%) | 214(23.88%) | 137(15.29%) | 420(46.88%) |  |
| DM |  |  |  |  | 0.76 |
| No | 120(13.39%) | 376(41.96%) | 227(25.33%) | 723(80.69%) |  |
| Yes | 29(3.24%) | 85(9.49%) | 59(6.58%) | 173(19.31%) |  |

Notes： Among the smokers, 0 means never smoking, 1 means occasional smoking, smoking cigarettes more than 4 times a week, but less than 1 cigarette per day on average, 2 means regular smoking, smoking cigarettes more than 1 cigarette per day, continuous or accumulative for 6 months. 3 represents heavy smoking, smoking more than 2 cigarettes a day for more than 6 months. Zero for non-drinking, 1 for occasional drinking, consuming about 10 grams of alcohol per serving, and 2 for regular drinking, exceeding about 10 grams of alcohol.
