# Supplementary material for: GLUT4 gene rs5418 polymorphism is associated with increased coronary heart disease risk in a Uygur Chinese population
Source: BMC Cardiovasc Disord. 2022 Apr 25;22:191. doi: 10.1186/s12872-022-02630-9 (PMC9036804; doi:10.1186/s12872-022-02630-9)
Supplement: Supplementary file 2 — Additional file 2: Figure S1. Haploview analysis for D' and r2 pairwise measures of LD between rs5418 and rs5435. D' values and confidence levels (LOD) are represented as black for D' = 1, LOD > 2; shades of pink for high D', LOD < 2; white for D' < 1, LOD < 2. r2 values are represented as black for r2 = 1, white for r2 = 0, with intermediate values for 0 < r2 < 1 indicated by shades of grey. The numbers within the squares represent the D' or r2 scores for pairwise LD. [file 12872_2022_2630_MOESM2_ESM.docx]

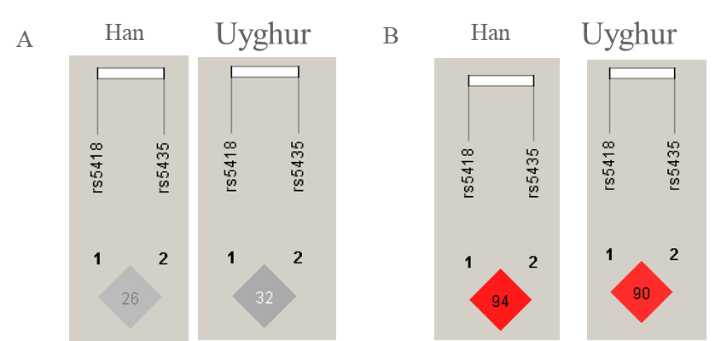


Figure S1. Haploview analysis for D' and r2 pairwise measures of LD between rs5418 and rs5435. D' values and confidence levels (LOD) are represented as black for D' = 1, LOD>2; shades of pink for high D', LOD<2; white for D'<1, LOD<2. r2 values are represented as black for r2 = 1, white for r2 = 0, with intermediate values for 0<r2 < 1 indicated by shades of grey. The numbers within the squares represent the D' or r2 scores for pairwise LD.
